# Supplementary material for: Mutations in PCYT2 disrupt etherlipid biosynthesis and cause a complex hereditary spastic paraplegia
Source: Brain. 2019 Oct 22;142(11):3382–97. doi: 10.1093/brain/awz291 (PMC6821184; doi:10.1093/brain/awz291)
Supplement: awz291_Supplementary_Data [file awz291_supplementary_data.zip › awz291-Suppl_data/Supplementary_Data4.zip!/Supplementary information 3 - fibroblast lipidomics.zip!/Fibroblast lipidomics/Controle vs Patient1/Statistical plots and data/BMP species/Boxplots all.pdf]

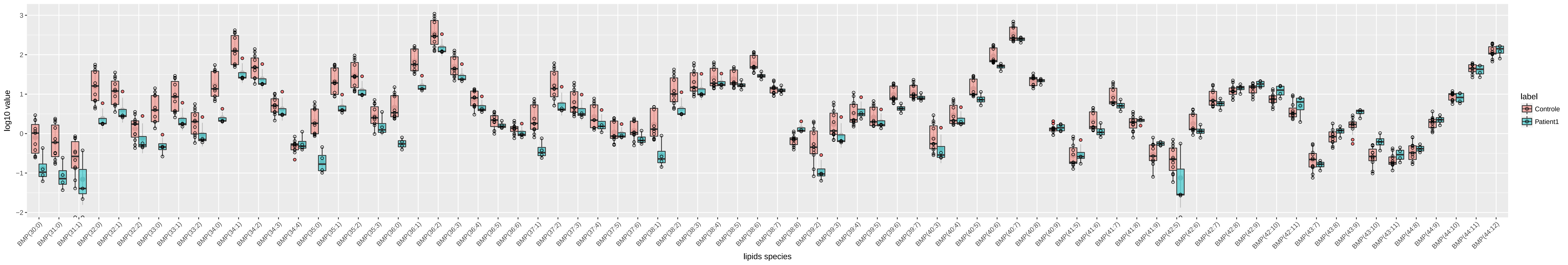
log10 value
3
2
1
0
−1
−2
BMP(30:0)
BMP(31:0)
BMP(31:1)
BMP(32:0)
BMP(32:1)
BMP(32:2)
BMP(33:0)
BMP(33:1)
BMP(33:2)
BMP(34:0)
BMP(34:1)
BMP(34:2)
BMP(34:3)
BMP(34:4)
BMP(35:0)
BMP(35:1)
BMP(35:2)
BMP(35:3)
BMP(36:0)
BMP(36:1)
BMP(36:2)
BMP(36:3)
BMP(36:4)
BMP(36:5)
BMP(36:6)
BMP(37:1)
BMP(37:2)
BMP(37:3)
BMP(37:4)
BMP(37:5)
BMP(37:6)
BMP(38:1)
BMP(38:2)
BMP(38:3)
BMP(38:4)
BMP(38:5)
BMP(38:6)
BMP(38:7)
BMP(38:8)
BMP(39:2)
BMP(39:3)
BMP(39:4)
BMP(39:5)
BMP(39:6)
BMP(39:7)
BMP(40:3)
BMP(40:4)
BMP(40:5)
BMP(40:6)
BMP(40:7)
BMP(40:8)
BMP(40:9)
BMP(41:5)
BMP(41:6)
BMP(41:7)
BMP(41:8)
BMP(41:9)
BMP(42:5)
BMP(42:6)
BMP(42:7)
BMP(42:8)
BMP(42:9)
BMP(42:10)
BMP(42:11)
BMP(43:7)
BMP(43:8)
BMP(43:9)
BMP(43:10)
BMP(43:11)
BMP(44:8)
BMP(44:9)
BMP(44:10)
BMP(44:11)
BMP(44:12)
lipids species
label
Controle
Patient1
